# Phospho-Regulation of Meiotic Prophase

**DOI:** 10.3389/fcell.2021.667073

**Published:** 2021-04-13

**Authors:** Funda M. Kar, Andreas Hochwagen

**Affiliations:** Department of Biology, New York University, New York, NY, United States

**Keywords:** meiosis, prophase, recombination, synapsis, checkpoint, kinase, phosphatase

## Abstract

Germ cells undergoing meiosis rely on an intricate network of surveillance mechanisms that govern the production of euploid gametes for successful sexual reproduction. These surveillance mechanisms are particularly crucial during meiotic prophase, when cells execute a highly orchestrated program of chromosome morphogenesis and recombination, which must be integrated with the meiotic cell division machinery to ensure the safe execution of meiosis. Dynamic protein phosphorylation, controlled by kinases and phosphatases, has emerged as one of the main signaling routes for providing readout and regulation of chromosomal and cellular behavior throughout meiotic prophase. In this review, we discuss common principles and provide detailed examples of how these phosphorylation events are employed to ensure faithful passage of chromosomes from one generation to the next.

## Introduction

The central function of meiosis is to produce haploid genomes that can be packaged into gametes for sexual reproduction. Going from a diploid germ cell progenitor to haploid meiotic products involves a modified cell division program, in which a single round of DNA replication is followed by two rounds of chromosome segregation, meiosis I and meiosis II. These two segregation phases separate homologous chromosomes and sister chromatids, respectively (Petronczki et al., 2003; Hunter, 2013). The spatiotemporal regulation of meiotic events is highly complex and this complexity is accompanied by rates of chromosome missegregation that are orders of magnitude higher than during mitosis. Errors in meiotic chromosome segregation account for the naturally high rate of spontaneous pregnancy loss in humans and are the main cause of chromosomal birth defects, including Down syndrome (Nagaoka et al., 2012; Geisinger and Benavente, 2016; Potapova and Gorbsky, 2017; Webster and Schuh, 2017).

The complexities of meiosis arise primarily from the need to faithfully identify and connect homologous chromosome pairs and to ensure their proper separation during meiosis I. Unlike sister chromatids, which are connected by sister chromatid cohesion from the moment they are synthesized, homologous chromosomes originate from different organisms (mom and dad). As a result, meiotic germ cells spend an inordinate amount of time and energy to properly identify and connect pairs of homologous chromosomes (Zickler and Kleckner, 2015, 2016). This process occurs after premeiotic DNA replication in a period called meiotic prophase and, in most organisms, involves the physical rewiring of homologous chromosomes by meiotic crossover recombination. Crossover recombination is important evolutionarily for creating new allele combinations but also has an important mechanical function during meiosis. Together with the existing sister chromatid cohesion, crossovers create physical links between homologous chromosome pairs that support proper chromosome alignment and separation during meiosis I (Hunter, 2015).

**FIGURE 1:**
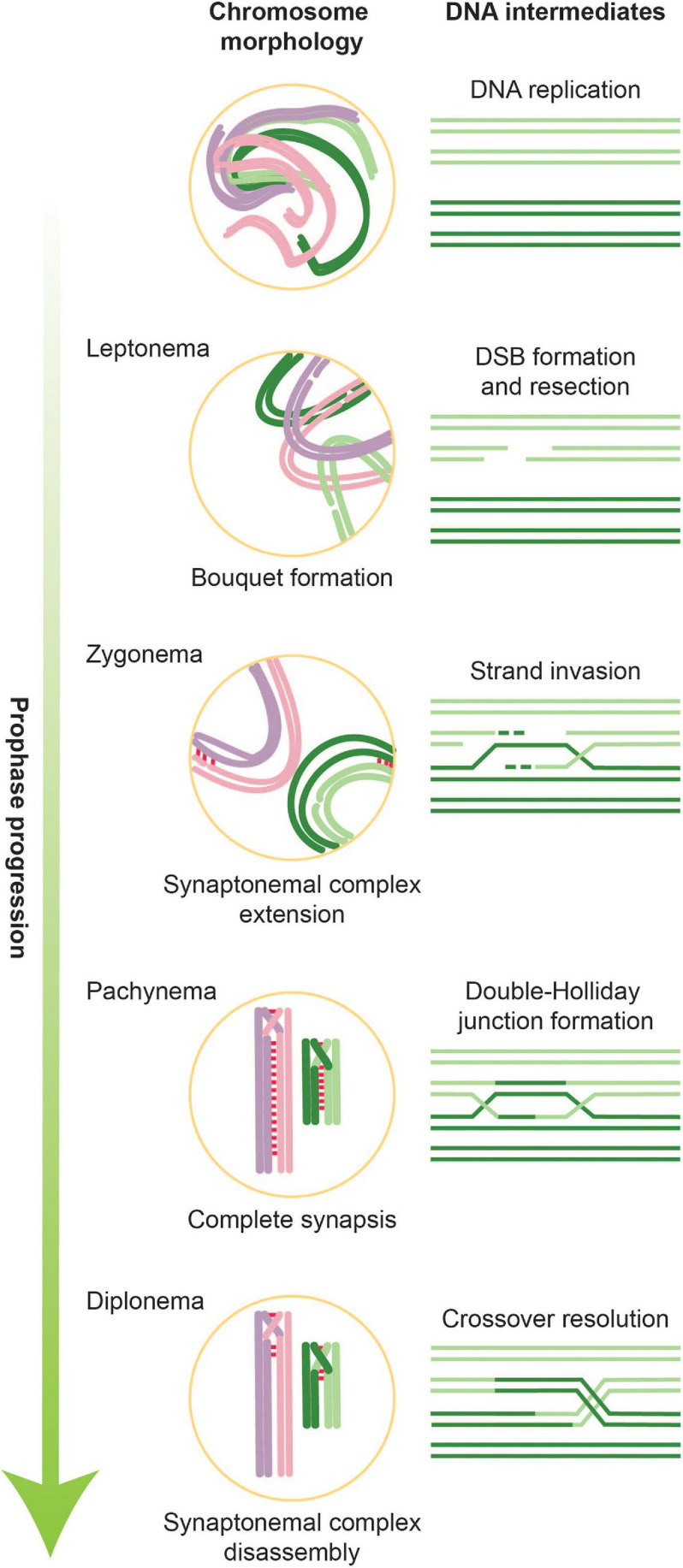
Overview of stages of meiotic prophase along with corresponding DNA intermediates. During leptonema, the first stage of meiotic prophase, chromatin condenses and forms attachments with the nuclear envelope. It is also during this stage that DSBs are introduced. In parallel with ongoing DNA repair, homologous chromosomes pair and formation of the SC is initiated. Partial SCs define the next meiotic stage, zygonema. When all chromosomes are synapsed along their axes, cells are in pachynema, which is when the bulk of double-Holliday junctions form. During late pachynema/early diplonema, double-Holliday junctions are resolved into crossovers and SC disassembly begins. Cells then exit meiotic prophase and prepare for the first meiotic division.

Crossover recombination involves the controlled introduction and repair of numerous DNA double-strand breaks (DSBs) (Figure 1). Meiotic DSBs are formed by SPO11, a conserved meiosis-specific enzyme related to topoisomerases that remains covalently attached to DSB ends and must be nucleolytically removed to allow repair (Lam and Keeney, 2015). Once removed, DSBs are resected to expose single-stranded DNA (ssDNA). These ssDNA tails provide the substrate for recombinases including RAD51 and DMC1, which scan the genome for homology and catalyze strand invasion of a donor duplex to initiate repair (Brown and Bishop, 2015). Depending on the processing of the resulting displacement loops, these intermediates are either stabilized and further processed to form crossovers, or they are dissolved after limited repair synthesis to yield non-crossover products (Hunter, 2015). To prevent genomic instability, DSBs must be repaired by the time cells initiate meiosis I.

Alongside these DNA-based events occur large-scale changes in chromosome architecture and dynamics (Zickler and Kleckner, 2015, 2016; Figure 1). Chromosomes assemble into longitudinally compacted arrays of chromatin loops that emanate from the axial element, a meiosis-specific nucleo-protein axis that dynamically adapts to ongoing transcription and recombination. As meiotic prophase progresses, axial elements of homologous chromosomes align to form the lateral elements of the synaptonemal complex (SC), a dynamic structure that progressively connects homologous chromosome pairs in a zipper-like arrangement (Zickler and Kleckner, 2015; Lake and Hawley, 2021). The chromosomal compaction and organization mediated by the SC are tightly coupled to the progression of crossover recombination and play numerous roles in controlling all stages of meiotic recombination. They also lead to gross morphological changes in chromosome architecture that underlie the cytologically defined stages of meiotic prophase, leptonema, zygonema, pachynema, and diplonema, which describe the progressive compaction and ultimate decompaction of prophase chromosomes. Alongside SC formation, chromosomes cluster with their telomeres in the nuclear envelope to form the telomere bouquet (Scherthan, 2001; Zickler and Kleckner, 2016). Telomeric attachment to the nuclear envelope also creates chromosomal linkages with the cytoplasmic cytoskeleton, which powers rapid chromosomal movements during pachynema.

The programmed formation of meiotic DSBs in the context of a highly dynamic genome creates a substantial hazard for genomic integrity. Meiotic checkpoints and surveillance mechanisms serve to safely navigate this developmental process and ensure that DSBs form at the right time and are appropriately repaired before cells initiate the meiotic divisions (MacQueen and Hochwagen, 2011; Subramanian and Hochwagen, 2014). These mechanisms ensure that DNA replication is largely complete before DSBs start to form, they help to locally downregulate DSB formation once a chromosome pair has initiated productive crossover recombination, and they stop DSB formation as cells exit prophase. In addition, signaling events suppress inappropriate repair patterns, control crossover maturation, and create dependent relationships between DSB repair and chromosome morphogenesis. Our understanding of this network has grown substantially as more connections are being uncovered and dissected at the molecular level, although available data suggest that this network is likely significantly more complex.

**FIGURE 2:**
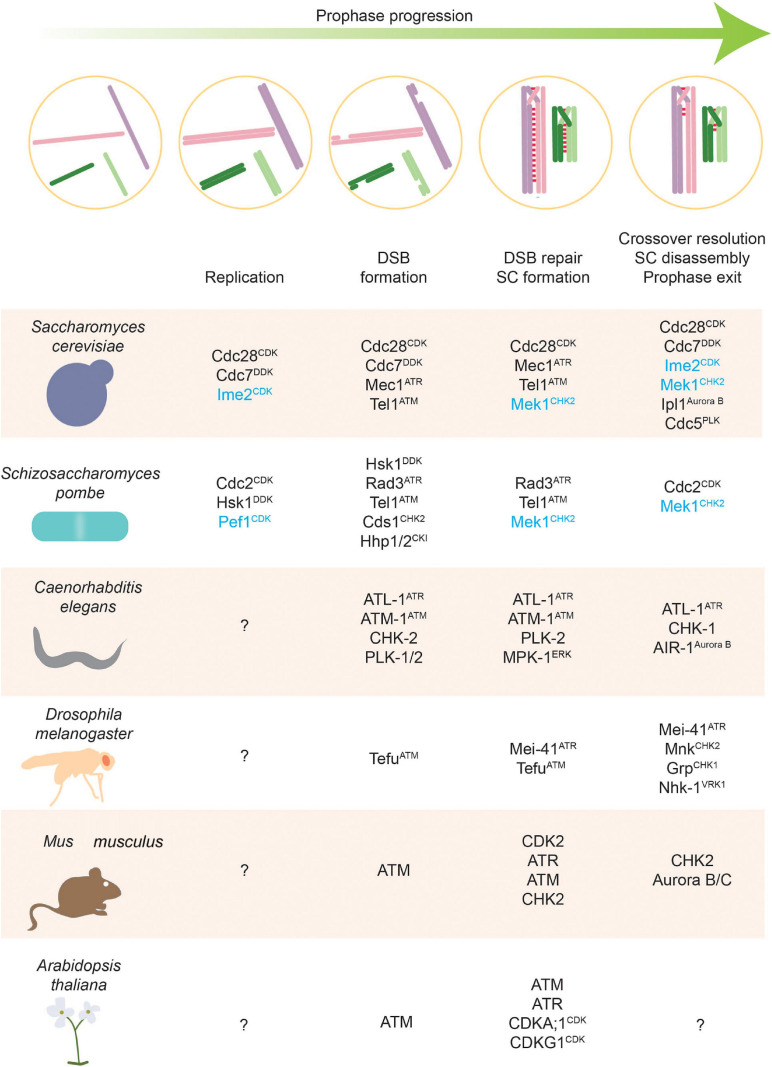
Stages of meiosis and kinases that regulate them. Kinases along with phosphatases are the main actors of phosphorylation-based regulatory control. This figure highlights kinases with defined roles in different meiotic stages. Meiosis-specific kinases are colored blue.

Dynamic protein phosphorylation has emerged as a major mediator of this signaling during meiotic prophase. Protein phosphorylation is a very versatile way of communication mediated by kinases and phosphatases. Kinases catalyze the transfer of the gamma-phosphate group of ATP onto suitable targets (in eukaryotes primarily the hydroxyl groups of serine, threonine, and tyrosine). Phosphatases reverse this process by hydrolyzing the resulting phosphoesters. Both classes of enzymes have important roles in the regulation of meiotic prophase, although research has primarily focused on the role of kinases (Figure 2). In part this bias arises because eukaryotic genomes encode more kinases than phosphatases (Smoly et al., 2017). However, phosphatases have crucial roles in controlling signaling dynamics and in executing large transitions during meiotic prophase.

## Functions of Phosphorylation

Research over the past two decades has greatly improved our understanding of how phosphorylation events regulate meiotic prophase. It has also revealed a number of recurrent regulatory modes that create dependencies, allow local decision-making, and integrate signals. We would like to highlight some of these modes before discussing the regulation of individual prophase processes in more detail.

### Creating Dependent Relationships

A key function of kinase signaling in meiotic prophase is the establishment of dependent relationships, whereby the ongoing activity of one process, such as the presence of DSBs, is communicated to other metabolically independent processes, such as centromere coupling or cell-cycle progression (Lydall et al., 1996; Falk et al., 2010). These dependent relationships can promote the co-occurrence of processes, or they can create a “wait” signal to ensure that one process is completed before the next process initiates (MacQueen and Hochwagen, 2011; Subramanian and Hochwagen, 2014). The creation of a wait signal is often referred to as a checkpoint (Hartwell and Weinert, 1989). Many of the known dependent relationships in meiotic prophase are established by a core kinase signaling network, consisting of the DNA-damage sensor kinases ATM and ATR and the transducer kinase CHK2 (Pereira et al., 2020). ATM and ATR sense protein-linked DNA ends and RPA-coated single-stranded DNA, respectively (Marechal and Zou, 2013; Awasthi et al., 2015). CHK2 gets activated by ATM/ATR and targets an additional set of substrates (Stracker et al., 2009). In meiotic prophase, all three kinases have an expanded substrate spectrum that includes numerous meiosis-specific proteins (Carballo et al., 2008, 2013; Falk et al., 2010; Kim et al., 2015; Penedos et al., 2015). In addition, the architecture of the signaling cascade appears to be rewired in multiple ways. All three kinases display altered (DSB-independent) modalities of activation in at least some organisms (Barchi et al., 2005; Bellani et al., 2005; Bhalla and Dernburg, 2005; Blanco-Rodríguez, 2012; Widger et al., 2018). In addition, several cell-cycle kinases, including Dbf4-dependent kinase (DDK) and Polo-like kinases (PLKs), have been tied into this network to establish dependencies (Clyne et al., 2003; Sasanuma et al., 2008; Sourirajan and Lichten, 2008; Wan et al., 2008; Labella et al., 2011; Murakami and Keeney, 2014; Nadarajan et al., 2017). On the other hand, several well-known mediators of canonical DNA-damage signaling, including the *Saccharomyces cerevisiae* adaptor protein Rad9^53BP1^ and the metazoan effector protein p53 appear to have less pronounced roles (Lydall et al., 1996; Odorisio et al., 1998; Murakami and Nurse, 1999; Ward et al., 2003; Ashley et al., 2004).

### Local Versus Global Signaling

The inherent asynchrony of DNA metabolism within the genome of each nucleus during meiotic prophase, including differences in the local timing of DNA replication, DSB formation, and repair kinetics, necessitates spatially restricted communication to coordinate processes at individual loci or on individual chromosomes. For example, DSB formation in yeast is locally licensed by DDK, which is thought to ride along with the DNA replication machinery and thus promote DSB formation specifically in DNA regions where replication is completed (Murakami and Keeney, 2014). Spatially constrained signaling is also inherent to ATM and ATR. Their damage dependency ensures that both kinases are principally active when tethered to DSB sites (Marechal and Zou, 2013; Awasthi et al., 2015; Paull, 2015) and helps establish local signaling hubs around DSB sites (Cimprich and Cortez, 2008; Blackford and Jackson, 2017). In mammalian spermatocytes, ATR is also activated independently of meiotic DSBs through the recruitment to unpaired chromosome axes (Keegan et al., 1996; Moens et al., 1999; Turner, 2015), thereby constraining kinase activity to specific chromosomal regions. An analogous situation occurs in *S. cerevisiae* where the meiotic CHK2-like transducer kinase, Mek1, depends on interactions with axis components to become active (Carballo et al., 2008). With kinase activity confined to these regions, it depends on the diffusibility of the respective kinase substrates whether signaling is locally constrained as seen for chromatin-associated phosphorylation events (Rogakou et al., 1999; He et al., 2020; Raina and Vader, 2020) or whether the signal is able to spread through the nucleus or the whole cell. Finally, dephosphorylation can also be spatially constrained. For example, meiotic cohesin is retained around centromeres during and after meiosis I due to localized activity of protein phosphatase 2A (PP2A) in these regions (Wassmann, 2013). These localized mechanisms allow signaling to occur independently at multiple locations despite their presence in a common nuclear space.

### Signal Amplification and Integration

A number of instances have been described in meiotic prophase where phosphorylation of one residue by an initiator kinase primes the protein for additional phosphorylation events mediated by another kinase. Such priming events can help amplify a signal by creating a larger region of phosphorylation (Wan et al., 2008; Falk et al., 2010; Chen et al., 2015). They also provide an opportunity to integrate multiple signals if the two kinases are differentially regulated. For example, DSB formation in *S. cerevisiae* requires priming phosphorylation of the DSB activator Mer2 by cyclin-dependent kinase (CDK) (Henderson et al., 2006; Sasanuma et al., 2008; Wan et al., 2008). This phosphorylation event creates a substrate recognition site for DDK, which subsequently phosphorylates additional sites (Sasanuma et al., 2008; Wan et al., 2008). Both of these phosphorylation events have to occur for DSB formation to initiate, but the two kinases are independently regulated. DDK is commonly observed as a responder to priming events, because it preferentially phosphorylates serines and threonines that are followed by residues with a negative charge, which can also be provided by phosphorylation. Several kinases, including DDK, CHK2, and PLK, also have phospho-binding domains that allow binding to specific phosphorylated targets and stimulate further phosphorylation. In addition, signal integration can also occur if two different kinases simply phosphorylate the same substrate. This pattern has emerged in a number of cases where a protein is phosphorylated by general cell cycle kinases with a wide spectrum of substrates, such as CDK, but also by a signal response kinase, such as ATM/ATR or CHK2 (Sopko et al., 2002; Callender et al., 2016; Chen et al., 2018). In these cases, phosphorylation by the cell-cycle kinase may communicate the appropriate cell cycle state and thus license the protein for regulation by the response kinase (or phosphatase).

## Control of Meiotic Prophase

This review intends to provide a solid overview of our current understanding of phospho-regulation of meiotic prophase in common model organisms of meiosis. However, given the wide spectrum of available examples, we apologize if space constraints prevented us from including all examples of this regulation.

## DNA Replication

Premeiotic S phase, though not formally part of meiotic prophase, is the first stage of meiosis-associated DNA metabolism and appears tightly integrated with subsequent prophase events. The phospho-regulation of premeiotic S phase has so far primarily been studied in *S. cerevisiae* and *Schizosaccharomyces pombe*, and, similar to mitotically proliferating cells, relies heavily on the activity of CDKs (MacKenzie and Lacefield, 2020). In *S. cerevisiae*, S-CDK (Cdc28^CDK^ bound to the cyclins Clb5 and Clb6) is essential for initiating premeiotic DNA replication (Stuart and Wittenberg, 1998; Benjamin et al., 2003). In *S. pombe*, the cyclin Cig2 bound to Cdc2^CDK^ similarly promotes premeiotic S phase, although *cig2* mutants only have a partial replication defect, because altered expression of later cyclins substitutes for the necessary CDK activity (Borgne et al., 2002; Malapeira et al., 2005). Cyclin gene expression patterns are consistent with CDKs also driving premeiotic S phase in mammals (Chotiner et al., 2019). One unusual feature of premeiotic S phase in both yeasts is the additional involvement of non-canonical CDKs. In *S. cerevisiae*, efficient S-CDK activation requires the meiosis-specific CDK2-like kinase Ime2, which promotes degradation of the CDK inhibitor Sic1 (Dirick et al., 1998; Benjamin et al., 2003; Sedgwick et al., 2006; Szwarcwort-Cohen et al., 2009). Like CDKs, Ime2 activation requires phosphorylation in its T-loop by the CDK-activating kinase Cak1 (Schindler et al., 2003). However, it does not require binding of a canonical cyclin (Honigberg, 2004). Ime2 likely has additional roles in activating DNA replication, because deletion of Sic1 does not rescue the DNA replication defects conferred by the absence of Ime2 (Dirick et al., 1998; Clifford et al., 2004). Although a large number of Ime2-dependent phosphorylation sites have been defined (Clifford et al., 2004; Holt et al., 2007), the relevant targets for premeiotic S phase activation remain to be determined. It is possible that the effect on S-phase activation occurs in part through Ime2’s role in promoting the meiotic gene expression program (Brush et al., 2012). A similar situation is observed in *S. pombe* where the CDK5-like kinase Pef1 promotes premeiotic DNA replication by inducing the expression of key replication factors (Matsuda et al., 2021). In both *S. cerevisiae* and *S. pombe*, timely activation of premeiotic DNA replication also requires DDK (Ogino and Masai, 2006; Valentin et al., 2006). In mitotically dividing cells, S-CDK and DDK phosphorylate numerous components of the pre-replicative complex to activate replication (Bell and Labib, 2016), although whether the same or additional targets become phosphorylated in meiosis remains to be determined.

## DSB Formation

### Connecting DSB Formation to DNA Replication

Meiotic DSB formation must be delayed until premeiotic DNA replication is largely complete because Spo11-induced DSBs on unreplicated DNA are difficult to repair and also interfere with the completion of DNA replication (Blitzblau and Hochwagen, 2013). Initiation of premeiotic DNA replication requires substantially lower levels of DDK activity than DSB formation (Wan et al., 2006), providing a basal mechanism to temporally separate the two processes. In addition, evidence suggests that formation of DSBs is coordinated with DNA replication in a local manner, as a delay in DNA replication in one region of a chromosome leads to a delay in DSB formation specifically in that region (Borde et al., 2000; Murakami and Keeney, 2014). However, the dependence of DSB formation on the completion of DNA replication is not strict, as both *S. cerevisiae* and *S. pombe*, can form DSBs in the absence of DNA replication (Hochwagen et al., 2005; Tonami et al., 2005; Ogino and Masai, 2006; Blitzblau et al., 2012). In *S. cerevisiae*, coordination between DNA replication and DSB formation involves phosphorylation of the Spo11-accessory protein Mer2. Mer2 is phosphorylated on S30 by CDK, which primes phosphorylation on S29 by DDK (Henderson et al., 2006; Sasanuma et al., 2008; Wan et al., 2008). Phosphorylation of these sites is required for formation of DSBs (Henderson et al., 2006; Sasanuma et al., 2008; Wan et al., 2008) and promotes localization of other components of the meiotic DSB machinery to chromosome axes (Panizza et al., 2011). Additional DDK-dependent phosphorylation events in the N-terminus of Mer2 also contribute to DSB formation, albeit more weakly (Sasanuma et al., 2008).

Double-strand break formation is thought to be connected to local completion of DNA replication through the activity of DDK. According to this model, DDK is recruited to replisomes and phosphorylates chromatin-bound Mer2 as replisomes pass through replicating DNA (Murakami and Keeney, 2014). Mer2 may not be the only target of this control mechanism as phospho-mimetic mutants of S29 or S30 cannot bypass the need for DDK or CDK for DSB formation (Wan et al., 2008), although it is also possible that the phospho-mimetic substitutions did not fully substitute for the lack of phosphorylation. The DSB factor Rec104 is phosphorylated during meiosis in a Spo11-independent manner and has a different electrophoretic mobility when DNA replication is blocked (Kee et al., 2004; Blitzblau and Hochwagen, 2013). Thus, it is possible that Rec104 provides a further link between replication and DSB formation. However, this model can still not explain how DSB formation can happen in the absence of DNA replication. A potential answer is suggested by the observations that DDK activity progressively increases in meiotic prophase (Matos et al., 2008; Wan et al., 2008) and that induced overexpression of DDK abrogates local differences in DSB timing (Murakami and Keeney, 2014). We therefore speculate that replication fork passage facilitates Mer2 phosphorylation, but that this barrier can also be overcome independently of replication once nuclear DDK activity is sufficiently high.

Whether and how DSB formation is linked to DNA replication in other organisms is less clear. Perhaps the most intriguing link comes from analysis of *Caenorhabditis elegans* CHK-2^CHK2^, which has emerged as a master regulator of meiotic recombination in this organism. *chk-2* mutants are defective in numerous aspects of meiotic recombination, including chromosome pairing and nuclear organization (MacQueen and Villeneuve, 2001; Oishi et al., 2001). CHK-2 is also essential for meiotic DSB formation through controlling chromatin association of the DSB regulator DSB-1 (Stamper et al., 2013). It has been proposed that CHK-2 activity provides a link between DNA replication and recombination (MacQueen and Villeneuve, 2001), although inhibiting premeiotic DNA replication compromises meiotic progression in both wild-type and *chk-2* mutants (MacQueen and Villeneuve, 2001). It also remains puzzling is how CHK-2 would be activated in this model because mutants lacking both canonical activators of CHK2 kinases (*atl-1^*ATR*^* and *atm-1^*ATM*^*) have less severe recombination defects than *chk-2* mutants (Li and Yanowitz, 2019). Perhaps other kinases substitute for ATM/ATR. Alternatively, CHK-2 activity may be primarily regulated at the level of gene expression (Mohammad et al., 2018).

### Role of the Replication Checkpoint

Research in *S. cerevisiae* and *S. pombe* has also revealed a mechanism that further delays DSB formation in response to persistent blocks in replication fork progression. In *S. cerevisiae*, the checkpoint kinases Mec1^ATR^ and Rad53^CHK2^ attenuate DDK activity in response to replication problems, thereby providing a mechanism to block DSB formation during replication stress (Blitzblau and Hochwagen, 2013). Mer2 phosphorylation happens independently of replication in *mec1*^ATR^ and *rad53*^CHK2^ mutants, suggesting that DDK’s association with replisomes is not essential for its ability to target and phosphorylate Mer2 (Blitzblau and Hochwagen, 2013). In addition to inhibiting DDK, replication stress controls DSB formation by downregulating Spo11 transcript levels (Blitzblau and Hochwagen, 2013), but how the replication checkpoint intersects with transcriptional regulation has not been investigated. In *S. pombe*, Rad3^ATR^ along with Cds1^CHK2^ is similarly required for preventing DSB formation in response to replication stress by downregulating expression of the transcription factor Mei4 (Tonami et al., 2005; Ogino and Masai, 2006). One key factor affected by this regulation is Mde2, which tethers the DSB machinery to the meiotic chromosome axis and is essential for DSB formation (Abe and Shimoda, 2000; Miyoshi et al., 2012, 2013). In addition, Mei4 downregulation leads to prolonged nuclear movement (Ruan et al., 2015). Whether similar regulation exists in mammals remains unclear. One potential candidate for checkpoint-dependent regulation is the protein ANKRD31, which interacts with the DSB factor REC114 (Boekhout et al., 2019; Papanikos et al., 2019). Mouse *Ankrd31−/−* mutants experience a delay in DSB formation along with other defects in recombination, including DSB patterning (Boekhout et al., 2019; Papanikos et al., 2019). ANKRD31 harbors 24 ATM/ATR consensus sites and thus has the potential to be a target for ATM/ATR-dependent regulation for DSB formation (Boekhout et al., 2019).

### Control of DSB Numbers

The number of meiotic DSBs per cell must be carefully titrated to ensure sufficient DSB formation for successful meiotic recombination while minimizing the risk of chromosome abnormalities associated with excessive DSB numbers. ATM mutants in various organisms exhibit increased DSB formation, suggesting a universal role for ATM in the regulation of DSB levels (Joyce et al., 2011; Lange et al., 2011; Checchi et al., 2014; Garcia et al., 2015; Mohibullah and Keeney, 2017; Fowler et al., 2018; Kurzbauer et al., 2021). In *S. cerevisiae* and *S. pombe*, ATM regulates DSB numbers by preventing double-cutting in proximity of DSBs (Garcia et al., 2015; Fowler et al., 2018). The activity of *S. cerevisiae* Tel1^ATM^ also limits DSB formation at a given locus to one DSB per quartet of chromatids (Zhang et al., 2011). Conversely, Mec1^ATR^ is suggested to promote DSB formation by blocking prophase exit until enough DSBs are made (Gray et al., 2013). Antagonistic roles of ATM and ATR in DSB formation have also been observed in *C. elegans* (Li and Yanowitz, 2019).

The definition of the relevant ATM substrates remains incomplete. In the protist *Tetrahymena thermophila*, the DSB factor Pars11 gets phosphorylated by ATR upon DSB formation (this organism lacks ATM) and is removed from chromatin and degraded (Tian and Loidl, 2018). Mutants lacking ATR during meiosis or expressing non-phosphorylatable versions of Pars11 exhibit elevated DSB numbers (Tian and Loidl, 2018), suggesting that Pars11 is a major mediator of feedback control in this organism. In *S. cerevisiae*, negative feedback of DSB formation is suggested to be partially achieved through the essential DSB factor Rec114, which gets phosphorylated by Tel1^ATM^/Mec1^ATR^ at multiple sites after DSB formation (Carballo et al., 2013). These phosphorylation events were found to reduce binding of Rec114 at DSB hotspots and reduce DSB levels (Carballo et al., 2013). However, altering Rec114 phosphorylation had little effect on DSB numbers in another study and did not recapitulate the increased DSB numbers or double cutting observed in ATM mutants (Garcia et al., 2015; Mohibullah and Keeney, 2017). It has been suggested that Rec114 might be only one of the targets of Tel1^ATM^, with each target contributing slightly to DSB-number regulation (Garcia et al., 2015; Mohibullah and Keeney, 2017). In *C. elegans*, the proteins DSB-1 and DSB-2 are required for DSB formation, and are suggested to be involved in a negative feedback regulation of DSB formation (Rosu et al., 2013; Stamper et al., 2013). DSB-1 and DSB-2 have clustered ATM/ATR phosphorylation sites, a feature that is also seen in other meiotic ATM/ATR targets like Rec114 (Carballo et al., 2013; Rosu et al., 2013; Stamper et al., 2013). Whether ATM/ATR function through these proteins to modulate DSB levels remains to be determined. Notably, feedback on DSB numbers in *C. elegans* also involves the polo-like kinases PLK-1 and PLK-2, which phosphorylate the SC protein SYP-4 on S269 (Nadarajan et al., 2017). This phosphorylation event takes place after a DSB site is designated as a crossover, connecting crossover designation to preventing further DSB formation in this organism (Nadarajan et al., 2017).

Other kinases also contribute to DSB formation. For example, casein kinase I (CKI) regulates DSB formation in fission yeast (Phadnis et al., 2015; Sakuno and Watanabe, 2015) by phosphorylating the meiosis specific cohesin subunit Rec11^STAG3^, which regulates loading of the DSB-promoting axis protein Rec10 (Phadnis et al., 2015; Sakuno and Watanabe, 2015). STAG3 is also phosphorylated in a *SPO11*-independent manner in mouse but the role of this phosphorylation has not been explored (Fukuda et al., 2012).

## Chromosome Dynamics

Protein phosphorylation has several key functions in controlling nuclear organization, chromosome movement, pairing, and synapsis in meiotic prophase.

### Nuclear Organization and Chromosome Movement

**FIGURE 3:**
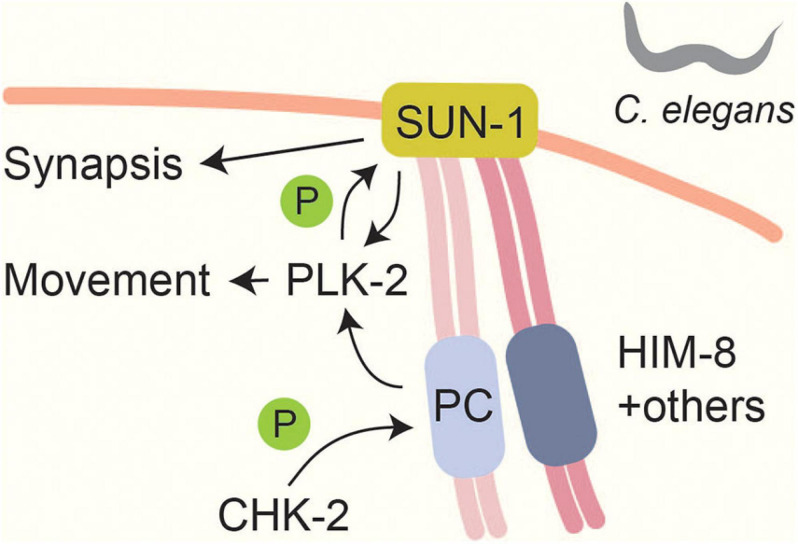
Linking chromosome movements and synapsis in *Caenorhabditis elegans*. A CHK-2 mediated signal localizes pairing centers (PCs) to the nuclear envelope. CHK-2 phosphorylates PC proteins like HIM-8 at PLK binding motifs (Kim et al., 2015). This phosphorylation leads to PLK-2 localization to PCs and promotes SUN-1 phosphorylation (Penkner et al., 2009; Harper et al., 2011; Labella et al., 2011). Phosphorylation of SUN-1 stabilizes PLK-2 at PCs and is thought to be part of a checkpoint that responds to synapsis and recombination defects (Woglar et al., 2013). SUN-1 phosphorylation promotes efficient SC formation (Woglar et al., 2013).

Meiotic chromosomes form connections with the nuclear envelope (Scherthan, 2001) and move to facilitate homolog pairing and resolution of chromosome entanglements (Zickler and Kleckner, 2016). These movements are mediated by SUN and KASH domain-containing proteins, which span the nuclear envelope and connect the chromosomes to the cytoskeleton (Hiraoka and Dernburg, 2009). In *C. elegans*, meiotic chromosome pairing and movement is initiated by the CHK-2-dependent phosphorylation and recruitment of chromosome-specific pairing proteins to subtelomeric pairing centers (Phillips and Dernburg, 2006; Kim et al., 2015; Figure 3). Some of the CHK-2-dependent phosphorylation sites (e.g., phosphorylation of HIM-8 on T64) occur in polo-box motifs and lead to the recruitment of PLK-2 (Kim et al., 2015). One output of this signaling is the CHK-2 and PLK-2 dependent phosphorylation of the SUN-domain protein SUN-1 (Penkner et al., 2009; Harper et al., 2011; Labella et al., 2011). SUN-1 phosphorylation stabilizes PLK-2 binding and promotes chromosome synapsis (Woglar et al., 2013), but there are likely additional substrates, because PLK-2 also promotes other processes, including chromosome pairing and movement, which were not disrupted by mutating SUN-1 phosphorylation sites (Harper et al., 2011; Labella et al., 2011; Woglar et al., 2013). Interestingly, CHK-2 itself appears to respond to phosphorylation, because its phospho-binding FHA domain is required for binding to pairing centers (Kim et al., 2015). However, the nature of the CHK-2 docking site remains to be determined.

Phospho-regulation also contributes to telomere tethering in mammals because mice lacking CDK2 or the atypical CDK activator Speedy A are defective in tethering telomeres to the nuclear envelope (Viera et al., 2015; Tu et al., 2017). CDK2 is required for the proper distribution of SUN1 in the nuclear envelope and can phosphorylate SUN1 *in vitro* (Viera et al., 2015), but whether this phosphorylation takes place *in vivo* and whether it is necessary for SUN1 distribution remains to be determined.

Finally, protein kinases also regulate the characteristic nuclear reorganization seen in several organisms during meiotic prophase. In *C. elegans*, CHK-2 and PLK-2 signaling organizes meiotic chromatin into a crescent shaped domain within the nucleus (MacQueen and Villeneuve, 2001; Harper et al., 2011; Labella et al., 2011), whereas the dramatic elongation of the meiotic micronucleus in the ciliate *T. thermophila* is induced by ATR in response to meiotic DSB induction (Mochizuki et al., 2008; Loidl and Mochizuki, 2009). In both instances the relevant kinase targets are currently unknown.

### Chromosome Pairing and Synapsis

As chromosomes undergo recombination at the DNA level, their axes pair up and, in many organisms, become stably aligned by the SC. This process is regulated by phosphorylation at multiple levels (Gao and Colaiácovo, 2018; Kim and Choi, 2019). In *Arabidopsis thaliana*, CDKA;1 phosphorylates the axis protein ASY1^HORMAD^, which is required for its recruitment to the axial element (Yang et al., 2020). Loss of another CDK, CDKG1, causes incomplete synapsis in male meiosis in a temperature-dependent manner (Zheng et al., 2014). In *S. cerevisiae*, reduced CDK activity also leads to defects in the formation of full-length SCs but the relevant phosphorylation targets remain to be determined (Zhu et al., 2010). In *C. elegans*, CDK-dependent phosphorylation of the SC protein SYP-1 primes recruitment of the PLK-2 to the SC (Sato-Carlton et al., 2018; Brandt et al., 2020). Interestingly, PLK-2 is prevented from binding to these primed sites until a chromosome undergoes crossover designation, whereupon CDK-1 dependent PLK-2 recruitment helps partition the holocentric chromosomes of *C. elegans* into short and long arms in preparation for the meiotic divisions (Sato-Carlton et al., 2018; Brandt et al., 2020).

Phosphorylation of SC proteins also helps break chromosomal interactions. In *S. cerevisiae*, the SC protein Zip1 connects pairs of centromeres independently of homology (Tsubouchi and Roeder, 2005). This association occurs separately from SC formation and is thought to provide a backup system for chromosomes that failed to undergo crossover formation (Obeso et al., 2014). Mec1^ATR^-dependent phosphorylation of Zip1 on S75 leads to the transient disruption of centromere coupling during meiotic prophase, presumably to enable homology-dependent pairing (Falk et al., 2010; Obeso et al., 2014). In the process, S75 phosphorylation primes Zip1 for phosphorylation on multiple additional residues that may amplify the effect of the initial phosphorylation event (Falk et al., 2010), but the nature and role of these additional events remains unknown.

Other kinases also impact SC formation and stability. In *C. elegans*, the ERK kinase MPK-1 is highly active in early-mid pachytene when it phosphorylates the SC proteins HTP-1 and SYP-2 (Lee et al., 2007; Nadarajan et al., 2016; Das et al., 2020). HTP-1 phosphorylation on S325 is essential for complete synapsis, as large stretches of chromosomes remain asynapsed in S325A mutants (Das et al., 2020). On the other hand, phosphorylation of SYP-2 prevents breakdown of SC on long chromosome arms (Nadarajan et al., 2016). A phospho-mimetic mutant of SYP-2 for this phosphorylation fails to disassemble its SC, similar to what is seen in mutants with sustained MPK-1 activity (Nadarajan et al., 2016). Pro-crossover proteins help to downregulate MPK-1 in late pachytene, thereby maintaining SC stability until after crossover formation has occurred (Nadarajan et al., 2016).

## DSB Repair and Crossover Formation

Crossover formation is a multi-step process with several decision points. Following break resection, DSBs must be targeted to the appropriate homologous repair template. A subset of DSBs is then designated to form joint molecules and double-Holliday junctions that are ultimately resolved as crossovers when cells exit meiotic prophase. Many of these processes and decision points are regulated by phosphorylation.

### Resection

Following DSB formation, SPO11 remains covalently linked to DSB ends. Processing of these protein-linked ends as well as the subsequent production of ssDNA ends by resection depends on the MRN complex and its activator CtIP. In *S. cerevisiae*, Sae2^CtIP^ tetramerization as well as its interaction with MRX^MRN^ is controlled by CDK-dependent phosphorylation of Sae2 S267, which is required for efficient resection (Huertas et al., 2008; Manfrini et al., 2010; Cannavo et al., 2018). Sae2 is also phosphorylated in a DSB-dependent manner by Mec1^ATR^ /Tel1^ATM^ (Baroni et al., 2004; Terasawa et al., 2008), but these modifications appear to be specifically required for Spo11 release from DSB ends and do not affect resection *in vitro* (Baroni et al., 2004; Terasawa et al., 2008; Cannavo et al., 2018).

### Suppression of Sister Repair

To encourage crossover recombination between homologous chromosomes, the use of the more readily available sister chromatid as a repair template must be suppressed. This suppression involves the combined action of the meiotic chromosome axes and specialized recombinase activities (Hollingsworth, 2010; Lao and Hunter, 2010; Humphryes and Hochwagen, 2014; Brown and Bishop, 2015; Rinaldi et al., 2017). In *S. cerevisiae*, phospho-regulation of repair template choice requires ATM/ATR-dependent phosphorylation of Hop1^HORMAD^ on T318 (Carballo et al., 2008). This phosphorylation event occurs specifically in the context of the axial element (Lin et al., 2010; Raina and Vader, 2020) and recruits the phospho-binding FHA domain of Mek1^CHK2^, resulting in the stabilization of phospho-T318 and Mek1^CHK2^ activation (Carballo et al., 2008; Chuang et al., 2012). Structural studies suggested that the FHA domain of Mek1^CHK2^ prefers hydrophobic amino acids at +2 and +3 positions from the phosphorylated residue (Xie et al., 2018), and the amino acids surrounding T318 fit this description. Whether any other phosphorylation events recruit Mek1^CHK2^ in a similar manner as Hop1^HORMAD^ is not yet known. Once activated, Mek1^CHK2^ phosphorylates at least two targets to suppress Rad51-mediated intersister repair. These include Rad54, a Rad51-interacting protein that stimulates Rad51 activity (Niu et al., 2009), and Hed1, a meiosis-specific protein that binds to Rad51 and displaces Rad54 (Callender et al., 2016). Mek1^CHK2^-dependent phosphorylation of Rad54 on T132 reduces its interaction with Rad51 (Niu et al., 2009), whereas phosphorylation of Hed1 T40 stabilizes Hed1 and thus downregulates Rad51 activity (Callender et al., 2016). Mek1^CHK2^ may phosphorylate additional meiotic factors to establish repair template choice because elimination of Rad54 phosphorylation and Hed1 together leads only to a two-fold decrease in homolog bias, whilst suppression of intersister repair is completely lost in the absence of Mek1^CHK2^ (Goldfarb and Lichten, 2010; Kim et al., 2010; Liu et al., 2014), but the relevant factors remain to be determined. ATM/ATR-dependent phosphorylation of the N-terminus of Rad51 may also contribute because it stabilizes Rad51 and is important for inter-homolog bias under certain conditions (Woo et al., 2020). A role for ATM in promoting interhomolog repair has also been suggested in *C. elegans* (Li and Yanowitz, 2019).

In several organisms, the suppression of sister repair is ultimately alleviated to promote repair of persisting meiotic DSBs. In *S. cerevisiae*, the recruitment of the AAA-ATPase Pch2^*TRIP13*^ to synapsing chromosomes leads to the removal of phospho-Hop1^HORMAD^-T318, partial inactivation of Mek1^*CHK2*^, and increased inter-sister repair (Subramanian et al., 2016). A transition of repair patterns during pachynema has also been reported for mouse spermatogenesis (Enguita-Marruedo et al., 2019). In *C. elegans*, ATM/ATR redundantly promote inter-sister repair in response to persistent DSBs (Garcia-Muse et al., 2019). A similar function has also been proposed for ATM in *Arabidopsis* (Yao et al., 2020). Notably, *Arabidopsis atr* mutants do not exhibit any meiotic phenotypes but exacerbate the meiotic defects of *atm* mutants, thus ATR likely plays a supporting role (Culligan and Britt, 2008; Yao et al., 2020).

One of the downstream phosphorylation targets of this response in *C. elegans* is the SC protein SYP-1, which is phosphorylated at six sites, none of which are in consensus ATM/ATR motifs, suggesting catalysis by other kinases (Garcia-Muse et al., 2019). Of note, one of these residues, T452, has also been implicated in crossover patterning and synapsis (Sato-Carlton et al., 2018). Puzzlingly, phospho-dead *syp-1 T452A* exhibit lower rates of embryo survival (∼60%) than *syp-1 6A* mutants (∼80%) (Sato-Carlton et al., 2018; Garcia-Muse et al., 2019), but whether mutation of the additional sites in the 6A mutant masks the effects of T452A remains to be investigated.

### Regulation of Crossover Designation

Once DSBs have encountered the homologous chromosome, repair intermediates that give rise to well-spaced (interfering) crossovers are stabilized by pro-crossover proteins. ATR mutants show defects in crossover patterning in multiple organisms (Brady et al., 2018; Li and Yanowitz, 2019; Shinohara et al., 2019). In *S. cerevisiae*, two key pro-crossover proteins, the SC protein Zip1 and the SUMO ligase Zip3^RNF212^ are both phosphorylated in this process. Phosphorylation on up to four consecutive serines in the C-terminus of Zip1 is important to promote crossover formation and to ensure efficient chromosome synapsis (Chen et al., 2015). One of these sites, S816, is phosphorylated by DDK in a DSB and Mek1^CHK2^-dependent manner (Chen et al., 2015). Since DDK uses prior phosphorylation events to recognize its targets, it is possible that there are other kinases that prime Zip1 at S817 or S818 to recruit DDK (Chen et al., 2015). As this region does not contain a canonical Mek1^CHK2^ motif, DDK-dependent phosphorylation might be primed by a different kinase than Mek1^CHK2^ or by Mek1^CHK2^ in a non-canonical manner (Chen et al., 2015). Zip3^RNF212^, which marks crossover sites, is phosphorylated in a Mec1^ATR^/Tel1^ATM^-dependent manner (Serrentino et al., 2013) and mutation of ATM/ATM consensus sites leads to a reduction of Zip3^RNF212^ recruitment to DSB sites and lower crossover frequency (Serrentino et al., 2013).

Phosphorylation of the SC protein SYP-1 also contributes to regulation of crossovers in *C. elegans*. Phosphorylation on SYP-1 T452 is required for wild-type crossover levels and patterning (Sato-Carlton et al., 2018). T452 is located in a polo-recognition motif and its phosphorylation promotes recruitment of PLK-2 to the short arms of chromosomes where phosphorylated SYP-1 is localized (Sato-Carlton et al., 2018).

Other phosphorylation events have been implicated in crossover formation in *S. cerevisiae*, although their exact mechanism and effect on crossover formation remain elusive. Like mutation of Zip3^RNF212^, mutation of phospho-sites in the meiotic cohesin Rec8 also causes a reduction in Zip3^RNF212^ foci (Yoon et al., 2016). In addition, Rfa2^RPA^ is phosphorylated at S122 by Mec1^ATR^ both in mitosis and meiosis, and a phosphomimetic mutant exhibits changes in crossover patterning in some intervals but not in others (Bartrand et al., 2006). However, how these phosphorylation events impact crossover-related phenomena has not been studied in detail.

One novel form of phospho-regulation was recently identified for the MutSγ complex, which contributes to formation of crossovers in many organisms (Hunter, 2015; Gray and Cohen, 2016). In *S. cerevisiae*, Msh4, one of the subunits of MutSγ, is phosphorylated in its N-terminus by DDK in a DSB-dependent manner (He et al., 2020). Phosphorylated Msh4 is enriched in the chromatin-bound fraction of Msh4 and depends on pro-crossover factors, suggesting that Msh4 phosphorylation occurs at sites of recombination (He et al., 2020). The activities of ATM/ATR and Mek1^CHK2^ are also required for this phosphorylation, although it is not clear whether their roles are direct (He et al., 2020). Intriguingly, Msh4 phosphorylation disrupts a degron sequence that would lead to proteasome-mediated degradation and thus may selectively stabilize Msh4 at crossover designated DSBs (He et al., 2020).

### A Role for CDKs

Cyclin-dependent kinases regulate crossover formation in mice and *Arabidopsis*. In *Arabidopsis*, reduction in CDKA;1 activity leads to a reduction in the number of crossovers and to changes in crossover distribution in certain genomic regions (Wijnker et al., 2019). In mice, CDK2 localizes to crossover sites (Ashley et al., 2001) and is required for completion of DSB repair (Viera et al., 2009). In hyperactive CDK2 mutants, the number of chromosomal foci of MHL1, a subunit of the pro-crossover MutLγ complex, increases although the number of crossovers does not change (Palmer et al., 2020). On the other hand, reduction of CDK2 activity blocks formation of crossovers (Palmer et al., 2020). How and through which targets CDKs promote crossover formation in these organisms remains to be answered, but they might be acting through similar pathways. Interestingly, a cyclin-related protein, COSA-1/Cntd1, marks crossover sites and is required for crossover formation in *C. elegans* and mice (Yokoo et al., 2012; Holloway et al., 2014; Gray et al., 2020). COSA-1/Cntd1 might partner up with CDK to promote crossover formation, although co-immunoprecipitation experiments in mice argue against a stable interaction between Cndt1 and CDK2 (Bondarieva et al., 2020; Gray et al., 2020).

### Regulation of Joint Molecule Resolution and Dissolution

In *S. cerevisiae*, Cdc5^*PLK*^ and its kinase activity are required for joint-molecule resolution into crossovers at the end of meiotic prophase (Clyne et al., 2003; Sourirajan and Lichten, 2008; Sanchez et al., 2020). Recent work has shed light on how different repair pathways are regulated by Cdc5^*PLK*^ to accomplish the appropriate processing of these repair intermediates.

Resolution of repair intermediates that were subject to crossover interference requires the interaction of Cdc5^PLK^ with the nuclease Exo1 (Sanchez et al., 2020). During meiosis, Exo1 forms a complex with the repair complex MutLγ, which is required for crossover formation and marks crossover sites (Zakharyevich et al., 2012). Unlike other examples involving PLK discussed in this review, the interaction between Exo1 and Cdc5^PLK^ does not depend on phosphorylation of Exo1 by another kinase (Sanchez et al., 2020). Since kinase activity of Cdc5^PLK^ is required for crossover formation independently of its interaction with Exo1, it is proposed that Cdc5^PLK^ phosphorylates yet unidentified targets to promote crossover resolution (Sanchez et al., 2020). The Mus81-Mms4 endonuclease complex also promotes joint-molecule resolution and is responsible for formation of non-interfering crossovers (de los Santos et al., 2003; Jessop and Lichten, 2008). Phosphorylation of Mms4 by Cdc5^PLK^ boosts Mus81-Mms4 activity in late pachynema, and this boost is required for timely resolution of repair intermediates (Matos et al., 2011). By contrast the endonuclease Yen1 is inhibited by Cdc28^CDK^ until the onset of meiosis II and likely serves only as a last resort to resolve persistent repair intermediates (Matos et al., 2011; Arter et al., 2018). Indeed, phosphorylation-resistant Yen1 is constitutively active, and this abnormal activation leads to early resolution of repair intermediates and aberrant crossover patterning (Arter et al., 2018).

The BLM helicase Sgs1 mediates the dissolution of joint molecules and is also regulated via phosphorylation by Cdc28^CDK^ (Grigaitis et al., 2020). Contrary to Yen1, Sgs1 activity is upregulated by Cdc28^CDK^-dependent phosphorylation, which is required for resolving some repair intermediates into non-crossovers (Grigaitis et al., 2020). In late pachynema, Sgs1 becomes hyper-phosphorylated by Cdc5^PLK^, and this phosphorylation is dependent on prior CDK phosphorylation (Grigaitis et al., 2020). Cdc5^PLK^-dependent phosphorylation of Sgs1 has been suggested to reduce its activity, thereby favoring joint-molecule resolution into crossovers in late pachynema (Grigaitis et al., 2020).

## The Pachytene Checkpoint

Many organisms delay meiotic progression in response to problems in meiotic DSB repair or defects in chromosome synapsis. These delays are similar to the cell-cycle delays observed in the canonical DNA-damage response and help to ensure that cells do not initiate the meiotic divisions with broken chromosomes (Mei et al., 2015; ElInati et al., 2020). The pachytene checkpoint employs the canonical DNA-damage sensor kinases ATR and ATM in most organisms (Hochwagen and Amon, 2006; Subramanian and Hochwagen, 2014). However, there are differences in downstream targets and effectors of this checkpoint.

**FIGURE 4:**
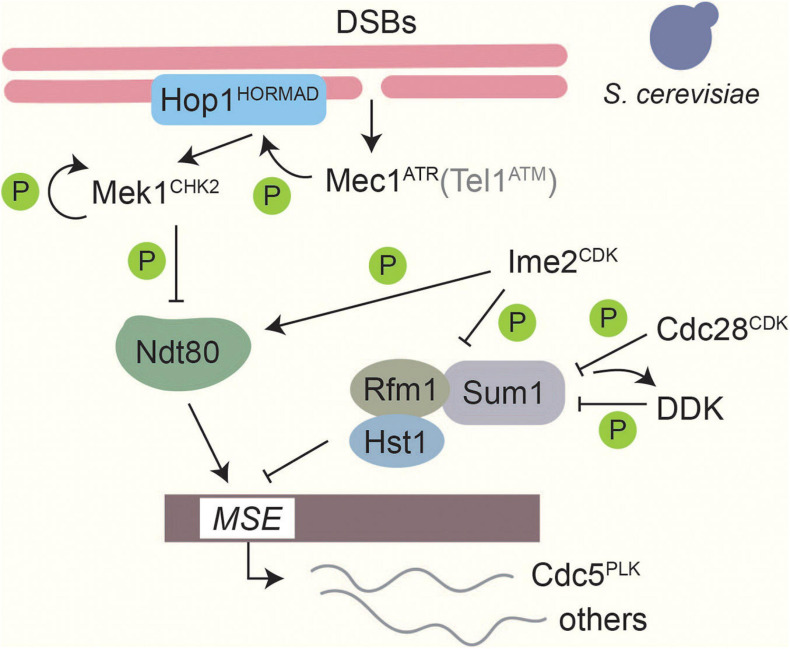
Phosphorylation-based regulation of middle gene transcription in *Saccharomyces cerevisiae*. Transcriptional control of middle genes in *S. cerevisiae* is under the control of several kinases. Formation of DSBs activates Mec1^*ATR*^, which in turn activates Mek1^*CHK2*^ through Hop1 phosphorylation and Mek1^*CHK2*^ autophosphorylation. Tel1^*ATM*^ contributes only weakly (Carballo et al., 2008). Ndt80 binding to middle sporulation elements (MSEs) is downregulated by Mek1^*CHK2*^ phosphorylation (Chen et al., 2018). Thus, when DSBs are present expression of middle sporulation genes is blocked. On the other hand, Ime2^*CDK*^-dependent phosphorylation promotes Ndt80 activity and facilitates competition with the Sum1 transcriptional repressor complex on MSEs (Sopko et al., 2002; Benjamin et al., 2003). Ime2^*CDK*^, along with Cdc28^*CDK*^ and Cdc7^*DDK*^, also phosphorylates Sum1 to promote dissociation of the repressor complex from MSEs (Lo et al., 2008; Sasanuma et al., 2008; Ahmed et al., 2009; Corbi et al., 2014).

In budding yeast, where this checkpoint is best understood, Mec1^ATR^/Tel1^ATM^ phosphorylate the axis protein Hop1^HORMAD^ at T318 as well as other sites (Carballo et al., 2008). Phosphorylated Hop1 recruits and activates Mek1^CHK2^ (Carballo et al., 2008) by mediating Mek1^CHK2^ dimerization and autophosphorylation (Niu et al., 2005, 2007). Phosphorylation of a different Mec1^ATR^ / Tel1^ATM^ site on Hop1, S298, is important to stabilize this interaction especially under checkpoint-activating conditions (Penedos et al., 2015). Mek1^CHK2^ phosphorylates a large number of downstream targets (Suhandynata et al., 2016), one of which is the meiosis-specific transcription factor Ndt80 (Prugar et al., 2017; Chen et al., 2018). Phosphorylation of Ndt80 attenuates its DNA binding and transcriptional activity and prevents expression of Cdc5^PLK^ (Chen et al., 2018), thereby blocking resolution of joint molecules and completion of meiotic prophase (Clyne et al., 2003; Sourirajan and Lichten, 2008; Figure 4). In *S. pombe*, Mek1^CHK2^ instead delays meiotic progression by phosphorylating the CDK-activating phosphatase Cdc25 and blocking its localization to the nucleus (Pérez-Hidalgo et al., 2003, 2008). Cdc25 promotes cell-cycle progression by removing the Wee1-dependent inhibitory phosphorylation on Cdc2^CDK^ Y15 (Pérez-Hidalgo et al., 2003, 2008). A role for tyrosine phosphorylation of CDK is also seen in *S. cerevisiae* (Leu and Roeder, 1999), although its regulation is not understood. In mice, ATM and CHK2 are also involved in the establishment of a checkpoint arrest in response to recombination defects (Bolcun-Filas et al., 2014; Pacheco et al., 2015; Rinaldi et al., 2017). Several chromosomal proteins are phosphorylated in a DSB-dependent manner in mice, including the Hop1 homologs HORMAD1/2 (Fukuda et al., 2012), but whether these phosphorylation events are important for checkpoint regulation remains to be investigated.

**FIGURE 5:**
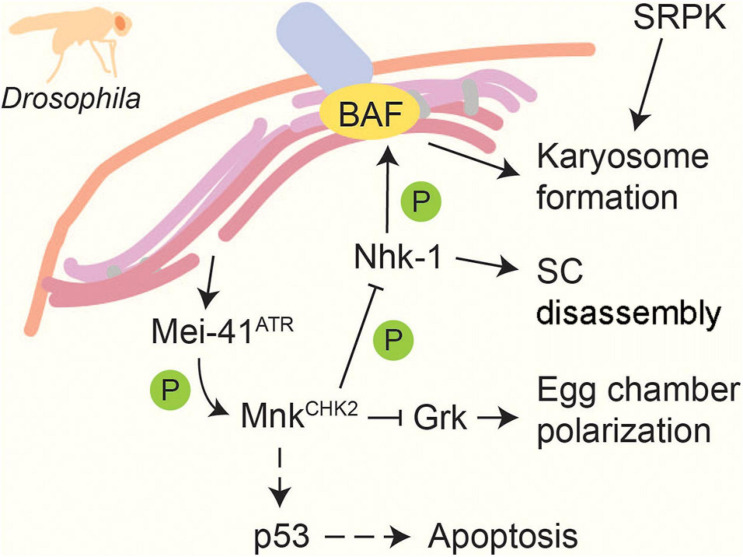
Recombination and meiotic progression in *Drosophila*. DSBs activate the DNA-damage sensor kinase Mei-41^*ATR*^, which in turn activates the effector kinase Mnk^*CHK2*^ (Ghabrial and Schüpbach, 1999; Abdu et al., 2002). Mnk^*CHK2*^ triggers p53-dependent apoptosis in response to persistent repair defects (Lu et al., 2010). Mnk^*CHK2*^ also suppresses translation of Gurken (Grk), which promotes egg chamber polarization (Abdu et al., 2002). In addition, Mnk^*Chk2*^ prevents meiotic progression by inhibiting Nhk-1, which normally prompts SC disassembly and induces karyosome formation by phosphorylating BAF (Ivanovska et al., 2005; Lancaster et al., 2007, 2010). Another kinase, SRPK, positively regulates karyosome formation in a Mnk^*CHK2*^- independent manner (Loh et al., 2012). Dashed lines indicate activation in response to persistent DNA damage signal.

In multicellular organisms, the pachytene checkpoint also activates apoptosis to eliminate germ cells that exhibit repair defects (Gartner et al., 2000; Barchi et al., 2005; ElInati et al., 2020). In *Drosophila melanogaster*, unrepaired breaks activate Mei-41^ATR^ and lead to the phosphorylation of Mnk^CHK2^ (Ghabrial and Schüpbach, 1999; Abdu et al., 2002; Figure 5). Brca2 is also involved in the activation of this checkpoint, presumably through its interaction with the checkpoint protein Rad9 (Klovstad et al., 2008). Mnk^CHK2^ in turn activates the pro-apoptosis protein p53 in response to persistent DSBs (Lu et al., 2010). Genetic experiments also identified a function for ATM and parallel roles for p53 and the p53-like regulator TAp63 in activating apoptosis during mouse spermatogenesis (Marcet-Ortega et al., 2017). In *C. elegans*, induction of apoptosis requires activation of the transducer kinase CHK-1 by ATL-1^ATR^ (Jaramillo-Lambert et al., 2010). The regulation in worms shows pronounced differences between hermaphrodites and males, possibly related to the inherent asynapsis of the monosomic X chromosome in *C. elegans* males (Gartner et al., 2000; Jaramillo-Lambert et al., 2010; Woglar et al., 2013). Moreover, the cell-death response varies depending on the mode of checkpoint activation (Ye et al., 2014).

Intriguingly, the pachytene checkpoint can also affect subsequent germline development. In *Drosophila*, this pathway reduces the levels of the developmental regulator Gurken, leading to impaired egg chamber polarization (Abdu et al., 2002), whereas the combined activity of CHK1 and CHK2 is required to reduce oocyte number and promote folliculogenesis in mice (Martínez-Marchal et al., 2020).

### The Role of Chromosome Architecture in Checkpoint Activation

Research in a number of organisms has led to the realization that the pachytene checkpoint senses more than just the accumulation of unrepaired DSBs. Although ATM/ATR clearly respond to DSB formation using canonical DNA damage response factors (Subramanian and Hochwagen, 2014), a number of observations indicate additional dependencies for full ATM/ATR activation. For example, the strong arrest response seen in *S. cerevisiae* mutants lacking the Dmc1 recombinase is weakened in mutants that also lack the second recombinase, Rad51, even though both mutants are severely defective in DSB repair (Shinohara et al., 2003). Similarly, the crossover factors MSH-4/5 and ZHP-3^RNF212^ are required for full checkpoint activation in *C. elegans*, raising the possibility that a downstream DNA repair intermediate helps activate the pachytene checkpoint (Silva et al., 2013). Perhaps most strikingly, research in mouse spermatocytes indicates that ATM/ATR are activated by unsynapsed chromosomal regions because the ATM/ATR-dependent γ-H2AX epitope is observed on unpaired X and Y chromosomes even in the absence of SPO11-induced DSBs (Mahadevaiah et al., 2001). Perhaps this activation is related to persistent SPO11-independent damage on asynaptic meiotic chromosomes as seen in mouse oocytes (Rinaldi et al., 2017).

Although the mechanisms of meiotic ATM/ATR activation remain enigmatic, available evidence suggests an important role for chromosome-structure proteins in stimulating full checkpoint activity. Components of the SC are required for checkpoint activation in *S. cerevisiae, C. elegans*, and mice (Xu et al., 1997; Daniel et al., 2011; Kogo et al., 2012; Wojtasz et al., 2012; Kim et al., 2015; Bohr et al., 2016), and altering the relative levels of individual SC components can lead to a severely disrupted checkpoint response without affecting other meiotic processes (Ontoso et al., 2013; Herruzo et al., 2016; Markowitz et al., 2017; Castellano-Pozo et al., 2020; Raina and Vader, 2020). Work in *S. cerevisiae, C. elegans*, and mouse has strongly implicated meiotic HORMAD proteins and their regulator Pch2/TRIP13 in this response. HORMAD proteins are very dynamic chromosomal constituents (Borner et al., 2008; Wojtasz et al., 2009; Subramanian et al., 2016) whose HORMA domain transitions between closed and unbuckled states, a behavior akin to the closed and open conformations of the essential spindle-checkpoint component MAD2 (Mapelli et al., 2007; Kim et al., 2014; West et al., 2018). The AAA+-ATPase Pch2/TRIP13 facilitates the formation of the unbuckled conformation (Ye et al., 2015; West et al., 2018).

Synapsis defects and recombination defects are signaled through a shared signaling network in several organisms (Hochwagen and Amon, 2006; Kim et al., 2015; Rinaldi et al., 2017). Early work in *C. elegans* suggested that Pch2^TRIP13^ specifically mediates the response to synapsis defects (Bhalla and Dernburg, 2005), and analyses in *S. cerevisiae* indicated the existence of Pch2^TRIP13^-dependent and Pch2^TRIP13^-independent checkpoint signaling (Wu and Burgess, 2006), which had been interpreted as separate synapsis and DNA damage sensing pathways (MacQueen and Hochwagen, 2011; Subramanian and Hochwagen, 2014). However, Pch2 responds to defects in DSB repair in *Drosophila* (Joyce and McKim, 2009), and *S. cerevisiae* mutants with defects in both synapsis and DNA repair show more severe Pch2^ TRIP13^-dependent delays than mutants that only affect synapsis (San-Segundo and Roeder, 1999; Humphryes et al., 2013; Herruzo et al., 2016), indicating that Pch2^TRIP13^ acts in the response to DNA repair defects.

Intriguingly, the mode of Pch2^TRIP13^ checkpoint function depends on its binding to the SC (Raina and Vader, 2020). A plausible model is that Pch2^TRIP13^-mediated accumulation of unbuckled Hop1^HORMAD^ in the nucleoplasm leads to a cell cycle delay whereas an accumulation of unbuckled Hop1^HORMAD^ on chromosomes leads to a checkpoint silencing (Raina and Vader, 2020). Consistent with this model Pch2^TRIP13^ executes its checkpoint-activating function when not bound to chromosomes (Herruzo et al., 2019), and Pch2^TRIP13^-dependent action on chromosomal Hop1^HORMAD^ silences Mek1^CHK2^-dependent signaling (Subramanian et al., 2016). The proposed action of Pch2^TRIP13^ on Hop1^HORMAD^ is reminiscent of the function of TRIP13 and MAD2 in the spindle checkpoint (Vader, 2015), and nicely explains why *pch2* mutations tend to alleviate arrests caused by synapsis defects but often strengthen arrests caused by DNA repair defects (Raina and Vader, 2020). What remains unclear is how Hop1^HORMAD^ conformation is interpreted to create the appropriate checkpoint response. Hop1^HORMAD^ phosphorylation is specifically detected on the chromosome-bound pool of Hop1^HORMAD^ (Herruzo et al., 2016; Raina and Vader, 2020), which polymerizes along chromosomes (West et al., 2019). By contrast Hop1 in the nucleoplasm is unphosphorylated and may be monomeric. Notably, Pch2 is necessary for preventing the phosphorylation of nucleoplasmic Hop1 (Lo et al., 2014), perhaps by keeping the HORMA domain unbuckled. However, which receptor interprets this form of Hop1 as a checkpoint signal is unknown. Pch2^TRIP13^ physically interacts with the phospho-binding BRCT domain of the DNA-damage response factor Xrs2^NBS1^, and this domain is required for pachytene checkpoint function (Ho and Burgess, 2011). Thus, perhaps the BRCT domain provides a mechanism to distinguish phosphorylated chromosomal and unphosphorylated non-chromosomal Hop1^HORMAD^.

## Formation of Nuclear Bodies

Several organisms form distinct nuclear bodies in the course of meiotic prophase, including the XY body in mammals and the karyosome in *Drosophila*. The formation of these structures is coordinated with meiotic progression and is regulated by protein phosphorylation.

### XY-Body Formation

Failure of chromosome synapsis leads to transcriptional silencing of asynaptic chromosomes in mammals, a phenomenon named meiotic silencing of unsynapsed chromatin (Schimenti, 2005; Turner et al., 2005). An example of this process in action is meiotic sex-chromosome inactivation, which is a regular occurrence during meiosis in spermatocytes (Schimenti, 2005; Turner et al., 2005). As mammalian X and Y sex chromosomes only share sequence homology in their pseudo-autosomal regions, they are mostly asynaptic during meiosis. Asynaptic sex chromosomes become transcriptionally silenced and form a cytologically detectable structure called the sex-body or XY-body through meiotic sex-chromosome inactivation (Solari, 1974; McKee and Handel, 1993).

The XY-body is strongly enriched for the canonical DNA damage mark, γH2AX (histone H2AX phosphorylated on S139), which localizes to the axes and chromatin loops of the asynaptic X and Y chromosomes in spermatocytes (Mahadevaiah et al., 2001). Knockout studies have shown that the H2AX histone variant is required for the establishment of the XY-body, although whether the γH2AX epitope is required has not been answered (Fernandez-Capetillo et al., 2003; Ichijima et al., 2011). γH2AX accumulation on the XY-body requires the DNA-damage response protein BRCA1, which recruits ATR to asynaptic chromosomes (Keegan et al., 1996; Turner et al., 2004; Bellani et al., 2005; Royo et al., 2013). The other canonical DNA-damage kinases ATM and DNA-PK are dispensable (Turner et al., 2004; Bellani et al., 2005). ATR-dependent phosphorylation of H2AX on the XY axes initially recruits the DNA-damage checkpoint factor MDC1, which mediates the expansion of γH2AX throughout the X and Y chromatin (Ichijima et al., 2011). Mutating a nearby phosphorylatable residue on H2AX, Y142, impairs expansion of the γH2AX signal similar to the loss of MDC1 (Abe et al., 2020). Interestingly, this failure in XY body formation caused a high level of ATR binding and accumulation of other DNA-damage marks on autosomes despite largely normal DSB levels (Abe et al., 2020). This observation led to the proposal that the XY body may provide a sink for DNA-damage signaling molecules, thereby allowing meiotic progression once repair has occurred (Abe et al., 2020). However, it is unclear why no such sink would be necessary during oogenesis, which involves similar levels of recombination but lacks a domain comparable to the XY body. DSB levels, as inferred from RAD51 foci, were elevated on the sex chromosomes in *H2ax-Y142A* but not *Mdc1*KO spermatocytes (Abe et al., 2020), which may point to a role for this residue in regulating DSB repair on asynaptic chromosomes, presumably by inter-sister repair. Interestingly, recruitment of the FHA domain of NBS1 to XY axes requires protein phosphorylation but not MDC1 (Zhang et al., 2020), and may thus be influenced by these histone marks.

In addition to ATR, sex chromosome silencing and XY body formation also requires CDK2 (Viera et al., 2009; Wang et al., 2014), which localizes to the XY body in its activated T160-phosphorylated form (Wang et al., 2014). The T160 phosphorylation enhances the interaction between γH2AX and CDK2 (Wang et al., 2014), but it is likely needed at later stages of XY body formation and gene silencing, because γH2AX localization to XY chromatin was unaffected in the CDK2 T160A mutant (Palmer et al., 2020).

### Karyosome Formation

Following recombination in *Drosophila* oocytes, chromosomes cluster into a spherical body called the karyosome, which subsequently nucleates acentrosomal spindle formation for the first meiotic division. A number of mutants with meiotic DSB-repair defects show abnormal karyosome formation (Ghabrial et al., 1998), which can be rescued by inactivation of the checkpoint kinase Mnk^CHK2^ (Abdu et al., 2002), indicating a dependent relationship between karyosome formation and meiotic recombination (Figure 5). Mnk^CHK2^ impairs the activity of the nucleosomal histone kinase Nhk-1, which phosphorylates the anchoring factor BAF to release chromosomes from the nuclear envelope (Lancaster et al., 2007, 2010). In addition, karyosome formation is regulated by the conserved kinase SRPK, which is required for heterochromatin clustering (Loh et al., 2012). A spherical chromosome assembly has also been reported in other organisms, including maturing human oocytes (Bogolyubov, 2018), but whether this structure is regulated in a similar manner remains to be determined.

## Meiotic Progression

Upon completion of meiotic recombination, meiocytes transition out of meiotic prophase by disassembling their SC and initiating the next steps of the meiotic program. These steps must be coordinated with meiotic recombination to prevent premature prophase exit or persistent SC during the meiotic divisions.

### SC Disassembly

Synaptonemal complex disassembly is regulated by phosphorylation in several organisms. SC disassembly in *Drosophila* requires Nhk-1 (Ivanovska et al., 2005), whose regulation by Mnk^CHK2^ helps tie SC disassembly to meiotic DNA repair (Lancaster et al., 2010; Figure 5). A similar dependence exists in *C. elegans*, where pro-crossover factors promote the downregulation of MPK-1, leading to the loss of SYP-2 phosphorylation and SC disassembly (Nadarajan et al., 2016). In *S. cerevisiae*, several kinases have been implicated in SC disassembly, including CDK, DDK, Cdc5^PLK^, and Ipl1^Aurora B^ (Sourirajan and Lichten, 2008; Jordan et al., 2009; Argunhan et al., 2017). PLK and Aurora B and C kinases are also required for desynapsis in mammals (Jordan et al., 2012; Wellard et al., 2020). SC disassembly in *S. cerevisiae* is tied to the successful completion of meiotic DSB repair through the control of Cdc5^PLK^ expression, which is maintained at a low level by proteasome-dependent degradation during meiotic prophase (Okaz et al., 2012) and whose expression is regulated at the transcriptional level by Mek1^CHK2^ (Tung et al., 2000; Pak and Segall, 2002b; Chen et al., 2018). However, the targets of Cdc5^PLK^ in promoting SC disassembly remain to be identified.

### Transcriptional Regulation of Prophase Exit

In *S. cerevisiae*, exit from meiotic prophase is marked by a strong shift in gene expression, which activates the “middle” genes necessary for completion of the meiotic divisions and spore maturation (Chu and Herskowitz, 1998). Expression of middle genes is under the control of the meiosis-specific transcription factor Ndt80 and the transcriptional repressor complex Sum1/Rfm1/Hst1 (Chu and Herskowitz, 1998; Xie et al., 1999; McCord et al., 2003), which compete for binding to a shared middle-sporulation element in the promoters of target genes. Three kinases are known to regulate Sum1 during meiosis (Figure 4). Ime2^CDK^ phosphorylates Sum1 at T306, which interferes with Hst1 binding and promotes Sum1’s dissociation from middle-sporulation elements (Moore et al., 2007; Ahmed et al., 2009). In addition, CDK^Cdc28^ phosphorylates Sum1 at multiple sites, including S379 and S512, which prime Sum1 to be phosphorylated by DDK (Lo et al., 2008; Sasanuma et al., 2008; Corbi et al., 2014). Phosphorylation promotes Sum1 removal from middle sporulation elements (MSEs) and is essential for the initiation of *NDT80* transcription (Pak and Segall, 2002a; Ahmed et al., 2009). Once Ndt80 production starts, Ndt80 can compete with Sum1 to bind to MSEs (Pierce et al., 2003). Ime2-dependent phosphorylation of Ndt80 promotes the ability of Ndt80 to bind DNA, and thus increases its activity (Sopko et al., 2002; Benjamin et al., 2003). Conversely, phosphorylation of Ndt80 by Mek1^CHK2^ reduces its ability to bind to DNA, and thus connects completion of DSB repair to exit from meiotic prophase (Chen et al., 2018; Hollingsworth and Gaglione, 2019).

## Reversal of Phosphorylation

Several phosphatases have been implicated in the regulation of meiotic prophase, although their functions in many cases remain poorly defined. Perhaps the best understood among these is protein phosphatase 4 (PP4), which acts to reverse ATR/ATM-dependent phosphorylation events similar to its function in the canonical DNA damage response (Nakada et al., 2008; Falk et al., 2010; Hustedt et al., 2015). Inactivation of PP4 leads to a number of meiotic defects in *S. cerevisiae* and *C. elegans*, including problems with chromosome synapsis and impaired crossover recombination (Falk et al., 2010; Sato-Carlton et al., 2014). However, so far only a small number of substrates have been identified in *S. cerevisiae*, including γH2AX, Hop1-T318, and Zip1-S75 (Falk et al., 2010; Chuang et al., 2012; Subramanian et al., 2016). PP4 appears to be continuously active during meiotic prophase and may thus require that ATR/ATM repeatedly phosphorylate the same substrates to maintain the DSB response (Falk et al., 2010). This arrangement would permit rapid removal of ATM/ATR-dependent phospho-marks once DSBs are repaired, and ATM and ATR are no longer active. In line with such dynamic regulation of phosphorylation events, binding of the FHA domain of Mek1^CHK2^ stabilizes the phosphorylation of Hop1-T318 (Chuang et al., 2012), possibly by blocking PP4 access. The rapid turnover of phosphorylated histone H2AV during meiotic prophase in *Drosophila* (Joyce et al., 2011) may result from a similar balance of kinase and phosphatase activities. In addition, continued PP4 activity may also help remove spurious or very transient phosphorylation events, as seen for the mitotic checkpoint kinase Rad53^CHK2^, which accumulates in a phosphorylated form in *pp4* mutants, but is not detectably phosphorylated in response to meiotic DSBs in wild-type cells (Cartagena-Lirola et al., 2008; Falk et al., 2010).

Protein phosphatase 1 (PP1) is another phosphatase implicated in the regulation of meiotic prophase, although its function may differ among organisms. In *C. elegans*, the PP1-interacting protein LAB-1 helps recruit PP1 to chromosomes to restrict the activity of AIR-1^Aurora B^ and promote SC assembly and DSB repair (Tzur et al., 2012). By contrast, PP1 activity is kept low in *S. cerevisiae* through binding of the FK506-binding protein Fpr3, which is important to maintain the activity of the pachytene checkpoint, as premature activation of PP1, or PP1 overexpression, allows DSB-repair mutants to enter the meiotic divisions (Bailis and Roeder, 2000; Hochwagen et al., 2005). Phosphatase inhibition is also important in *Drosophila* where overexpression of Wrd, a B56 subunit of protein phosphatase 2A, leads to delayed assembly and precocious disassembly of the SC, as well as karyosome defects (Barbosa et al., 2021). In this case, low Wrd levels in meiotic prophase depend on the ubiquitin ligase SCF (Barbosa et al., 2021), which presumably targets Wrd for proteasome-mediated degradation. Disruption of PP2A-Cdc55 has also been shown to affect premeiotic DNA replication and interhomolog recombination in *S. cerevisiae* (Nolt et al., 2011). Finally, the transient activation of the phosphatase LIP-1 is thought to counteract the activity of MPK-1, to create a window for damage-dependent germ cell apoptosis in *C. elegans* (Hajnal and Berset, 2002; Rutkowski et al., 2011).

## Challenges Ahead

Research over the past two decades has revealed many profound roles for protein phosphorylation in coordinating the processes of meiotic prophase. However, the rate at which new signaling connections are reported has not slowed, indicating that there are still many regulatory connections that remain to be uncovered before we can gain a systematic understanding of this impressive signaling network. The extent of the unknown is highlighted by a recent phospho-proteomic study, which identified a large number of novel phosphorylation events that were differentially regulated between wild-type and *mek1*^CHK2^ mutants in *S. cerevisiae* (Suhandynata et al., 2016). Although this study also identified many events that resulted from premature prophase exit in *mek1* mutants, a substantial number of novel phosphorylation events of unknown function could be attributed to Mek1 based on consensus sequences. Further targeted and stage-specific proteomic analyses therefore promise to provide a wealth of new information on the phospho-proteome of meiotic prophase.

With identification of phosphorylation sites becoming easier, pinpointing which ones are functionally important will be the difficult question to answer. Kinases can have somewhat promiscuous activity leading to “accidental” phosphorylation events (Levy et al., 2012). These accidental events can be influenced by several factors, including high concentration of a target protein in the proximity to the kinase (Levy et al., 2012). It has been suggested that integration of absolute protein abundance data with phosphorylation data or using stoichiometry of phosphorylation can help prioritize sites for characterization (Levy et al., 2012). However, even mutation of high-confidence sites frequently fails to yield an easily observable phenotype. For example, γH2AX and Mek1-dependent phosphorylation of histone H3 T11 are well-characterized chromatin marks that nevertheless appear to be dispensable for meiotic prophase in *S. cerevisiae* (Shroff et al., 2004; Kniewel et al., 2017). Some phosphorylation events may be unnecessary under standard laboratory conditions but have functions in specific environments or mutant situations. Others may have redundant activities. For example, in *S. cerevisiae* the cohesin Rec8 is phosphorylated at many sites, and the general level of phosphorylation rather than phosphorylation of any individual site is required for function (Brar et al., 2006). Such multi-site phosphorylations can mediate progressive charge accumulation or activate multiple low affinity sites to establish thresholds rather than switches, which can also be established by single phosphorylation events (Nash et al., 2001; Gunawardena, 2005; Salazar and Höfer, 2009). However, in the case of the abundantly phosphorylated *S. cerevisiae* axis protein Red1, even large-scale mutational sweeps of phosphorylation sites have failed to yield a detectable phenotype (Lai et al., 2011).

Evolutionary conservation is one of the predictors for functional importance of phosphorylation sites (Beltrao et al., 2012; Studer et al., 2016). Implementation of this predictor for meiotic phosphorylation events may be challenging as many meiotic proteins have very little sequence conservation across species (Cole et al., 2010; Kumar et al., 2010). In line with this, there are several examples for phosphorylation events that are important for meiosis but whose sites are not well conserved. These include Hop1 T318, which is essential for meiosis in *S. cerevisiae* but only conserved in yeasts and plants (Carballo et al., 2008) and Mer2 S30, which is absolutely required for formation of DSBs in *S. cerevisiae* but only conserved in yeasts (Wan et al., 2008). Even so, the general mechanisms by which these phosphorylation events act may be conserved even when exact sites are not. For example, mouse HORMAD1 and HORMAD2 contain S/TQ sites, and HORMAD1 is phosphorylated in an ATM/ATR dependent manner like Hop1 (Fukuda et al., 2012). It has been suggested that phosphorylation events that promote interactions through phosphopeptide binding domains (as is the case for Hop1 T318) may evolve more rapidly and may be less dependent on exact location for their function, while phosphorylation events that promote conformational changes may be more conserved (Holt et al., 2009). Similarly, clusters of target sites may show functional redundancy and thus, conservation of some of these sites may be enough to maintain function without a requirement for conservation of the exact position (Holt et al., 2009; Freschi et al., 2014).

A better understanding of phospho-regulation of meiotic prophase will also require a more detailed analysis of the spatial relationships of phosphorylation events. A number of experiments have demonstrated the power of phospho-specific antibodies in dissecting regulatory connections in *C. elegans* and other organisms (Carballo et al., 2008; Penkner et al., 2009; Labella et al., 2011; Woglar et al., 2013; Herruzo et al., 2016; Subramanian et al., 2016; Nadarajan et al., 2017), although the use of these reagents has often been limited to cytological analyses of fixed samples. Using the same antibodies in ChIP-seq experiments could provide complementary information at high genomic resolution, but this experimental route remains comparatively underused. In addition phospho-specific nanobodies and FRET-based kinase sensors (Oldach and Zhang, 2014; Traenkle and Rothbauer, 2017) may provide an opportunity to follow *in vivo* phospho-dynamics, which remains a key frontier in understanding the regulation of meiotic prophase. Finally, it will also be important to understand the crosstalk between protein phosphorylation with other signaling modes and protein modifications, including ubiquitylation and sumoylation, which are abundantly present in meiotic prophase (Cheng et al., 2006; Watts and Hoffmann, 2011; Nottke et al., 2017; Rao et al., 2017; Bhagwat et al., 2021), and to integrate nuclear events with regulation outside of the nucleus. For example, B-type cyclins in *S. cerevisiae* are translationally regulated by phosphorylation of an RNA-binding protein (Berchowitz et al., 2015; Carpenter et al., 2018) and mitochondrial localization is controlled by Ime2 kinase (Sawyer et al., 2019). Thus, notwithstanding the impressive progress in understanding the phospho-regulation of meiotic prophase, much remains to be discovered about this intricate signaling network that ensures the faithful passage of genetic material from one generation to the next.

## Author Contributions

FMK and AH performed the research and wrote the manuscript. Both authors contributed to the article and approved the submitted version.

## Conflict of Interest

The authors declare that the research was conducted in the absence of any commercial or financial relationships that could be construed as a potential conflict of interest.
